# “The path is smooth that leadeth on to danger”: caffeine and psychosis

**DOI:** 10.1192/bjo.2021.505

**Published:** 2021-06-18

**Authors:** Isabel Ganhao, Goncalo Marinho, Afonso Paixa, Miguel Trigo

**Affiliations:** Centro Hospitalar Psiquiatrico de Lisboa

## Abstract

**Aims:**

To review literature on the importance of caffeine intake with regard to psychosis. The need for intervention with regard to caffeine intake hinges on effectively recognizing potential risks.

**Background:**

Caffeine is the most widely consumed psychoactive substance worldwide and as such is generally considered acceptable but as a competitive adenosine antagonist, it affects dopamine transmission. Patients with serious mental illness are known to have higher caffeine intakes than the general population. The hierarchy of needs for this patient population is complex, frequently leaving the intake of caffeine under the radar of clinical priorities.

**Method:**

PubMed and Google Scholar search for caffeine/coffee and psychosis/schizophrenia

**Result:**

Of the 43 articles that were considered relevant for clinical practice, caffeine consumption was associated with 1) appearance of psychotic symptoms and episodes (caffeine-induced psychosis) and chronic psychosis in high intake 2) exacerbation of psychosis in schizophrenic patients even in lower intakes, 3) treatment resistance possibly due to interference with antipsychotics (ex. clozapine), 4) abuse and addiction, 5) comorbidity with tobacco smoking and other addictions. Caffeine in low doses was associated with ameliorating cognitive and extrapyramidal side-effects of medication and as a potential treatment strategy for treatment-resistant schizophrenia.

**Conclusion:**

Caffeine consumption may have a greater impact on psychotic symptoms and episodes than is recognized with negative effects outweighing any potential benefits. Greater awareness of the necessity to quantify caffeine intake and implementation of interventions to curb intake may contribute to better quality of care of serious mental illness. Further research is warranted.

